# Supplementary material for: Heterologous Expression of Poplar WRKY18/35 Paralogs in Arabidopsis Reveals Their Antagonistic Regulation on Pathogen Resistance and Abiotic Stress Tolerance via Variable Hormonal Pathways
Source: Int J Mol Sci. 2020 Jul 30;21(15):5440. doi: 10.3390/ijms21155440 (PMC7432504; doi:10.3390/ijms21155440)
Supplement: Supplementary file 1 [file ijms-21-05440-s001.pdf]

Supplementary Materials

Table 1. The list of primers used in real-time quantitative PCR reactions.

Gene Name	Accession Number	Primer Sequences (5'→3')
AtPR3	AT3G12500	FP-1: TGACTTACTCAACAACCCTGAC
		RP-1: CGTAATCACTCCATAACCCG
AtPDF1.2	AT5G44420	FP-2: GCTTCCATCATCACCCTTATC
		RP-2: GCTGGGAAGACATAGTTGC
AtVSP2	AT5G24770	FP-3: TCATACTCAGTGACCGTTGG
		RP-3: GGTATCCTCAACCAAATCAGC
AtPR1	AT2G14610	FP-4: CAGTGAGACTCGGATGTGC
		RP-4: TATGATGCTCCTTATTGAAATAC
AtPR2	AT3G57260	FP-5: GCAGCATTGGAGAAATCAG
		RP-5: TCTCATCGAACATAGCGAAT
AtPR5	AT1G75040	FP-6: GCTACGCTTATGACGACGAAAC
		RP-6: CATAGTCATCAGCTTTCCTTAT
AtNPR1	AT1G64280	FP-7: GCTTGCGGAGAAGACGACAC
		RP-7: GGCAAGAGTCTCACCGACG
AtRD29A	AT5G52310	FP-8: GATTAAAGGATGGTTCGGTG
		RP-8: CTTAAAGCTCCTTCTGCACC
AtABO3	AT1G66600	FP-9: GGTGTTACTATCGTTGTGCC
		RP-9: CGCATAACCGATTCAGATAC
AtABI4	AT2G40220	FP-10: TGGGACCTCTATGTTATGCCC
		RP-10: TGATGAAACGAAGTCGAATTAG
AtABI5	AT2G36270	FP-11: TGTCCACTCTAAACAAACAATAGG
		RP-11: TCACAGGGAACACTAGTAAAGC
AtDREB1A	AT4G25480	FP-12: CGTTTCAGGATGAGATGTGTG
		RP-12: CTTCATGATTATGATTCCACTG
AtRD22	AT5G25610	FP-13: GCGGTATGCCACAAGAAC
		RP-13: ACTCTTATATGGAAACCACGC
